# Activation of amylin receptors attenuates alcohol‐mediated behaviours in rodents

**DOI:** 10.1111/adb.12603

**Published:** 2018-02-06

**Authors:** Aimilia Lydia Kalafateli, Daniel Vallöf, Elisabet Jerlhag

**Affiliations:** ^1^ Department of Pharmacology, Institute of Neuroscience and Physiology The Sahlgrenska Academy at the University of Gothenburg Gothenburg Sweden

**Keywords:** addiction, food intake, gut–brain axis, mesolimbic dopamine system, reward, salmon calcitonin

## Abstract

Alcohol expresses its reinforcing properties by activating areas of the mesolimbic dopamine system, which consists of dopaminergic neurons projecting from the ventral tegmental area to the nucleus accumbens. The findings that reward induced by food and addictive drugs involve common mechanisms raise the possibility that gut–brain hormones, which control appetite, such as amylin, could be involved in reward regulation. Amylin decreases food intake, and despite its implication in the regulation of natural rewards, tenuous evidence support amylinergic mediation of artificial rewards, such as alcohol. Therefore, the present experiments were designed to investigate the effect of salmon calcitonin (sCT), an amylin receptor agonist and analogue of endogenous amylin, on various alcohol‐related behaviours in rodents. We showed that acute sCT administration attenuated the established effects of alcohol on the mesolimbic dopamine system, particularly alcohol‐induced locomotor stimulation and accumbal dopamine release. Using the conditioned place preference model, we demonstrated that repeated sCT administration prevented the expression of alcohol's rewarding properties and that acute sCT administration blocked the reward‐dependent memory consolidation. In addition, sCT pre‐treatment attenuated alcohol intake in low alcohol‐consuming rats, with a more evident decrease in high alcohol consumers in the intermittent alcohol access model. Lastly, sCT did not alter peanut butter intake, blood alcohol concentration and plasma corticosterone levels in mice. Taken together, the present data support that amylin signalling is involved in the expression of alcohol reinforcement and that amylin receptor agonists could be considered for the treatment of alcohol use disorder in humans.

## Introduction

Alcohol use disorder is a co‐morbid and highly prevalent disorder, causing severe health problems for both the society and the individual (Grant *et al*. 2015). However, existing pharmacotherapy has shown limited efficacy (Anton *et al*. 2006); therefore, further investigation of possible alcohol neurochemical targets could lead to new pharmacological interventions (Heilig & Egli 2006; Litten *et al*. 2012). Alcohol targets components of the mesolimbic dopamine system (Larsson & Engel 2004; Vengeliene *et al*. 2008; Soderpalm, Lof, & Ericson 2009), which is involved in the expression of its reinforcing properties (Adinoff 2004). Beyond the mesolimbic dopamine system, which includes dopamine projections from the ventral tegmental area (VTA) to nucleus accumbens (NAc) (Koob & Bloom 1988; Kelley & Berridge 2002), recent studies show that alcohol activates the cholinergic inputs from the laterodorsal tegmental nucleus (LDTg) (Larsson *et al*. 2005).

Emerged evidence suggests that feeding and reward behaviours share common complex neurochemical mechanisms and that these are mediated by the mesolimbic dopamine system (Rada, Mark, & Hoebel 1998; Hoebel *et al*. 1999; Addolorato *et al*. 2006; Jerlhag *et al*. 2006; Abizaid *et al*. 2011; Dickson *et al*. 2011; Leggio *et al*. 2011; Jerlhag *et al*. 2011a; Edwards & Abizaid 2016). More specifically, gut–brain peptides, which have been traditionally known to regulate food intake and energy balance (Ahima & Antwi 2008), seem to play a pivotal role in mediating the reinforcing properties of alcohol and other drugs of abuse (Jerlhag *et al*. 2010; Abizaid *et al*. 2011; Jerlhag & Engel 2011; Clifford *et al*. 2012; Egecioglu, Engel, & Jerlhag 2013a; Suchankova *et al*. 2013a; Engel & Jerlhag 2014; Vadnie *et al*. 2014; Vallof *et al*. 2016c). Notably, ghrelin, glucagon‐like peptide‐1 and neuromedin U have been shown to alter alcohol‐induced reward phenotypes by acting on the mesolimbic dopamine system (Kraus *et al*. 2005; Leggio *et al*. 2011; Jerlhag *et al*. 2011b; Landgren *et al*. 2012; Suchankova *et al*. 2013b; Leggio *et al*. 2014; Vallof *et al*. 2016a; Vallof *et al*. 2016b). Other hormones, for example, amylin, have been recently studied for their role to control energy balance through gut–brain axis regulation (Reda, Geliebter, & Pi‐Sunyer 2002; Lutz 2009). Amylin seems to regulate food intake through central mechanisms, by acting in areas of the mesolimbic dopamine system such as the VTA and LDTg (Mietlicki‐Baase *et al*. 2015b; Reiner *et al*. 2017), identifying it an interesting candidate for exploring its potential reward‐regulating properties.

Amylin is a 37‐amino acid hormone, co‐secreted with insulin in the pancreatic β‐cells (Westermark, Andersson, & Westermark 2011), and its physiological role includes insulin secretion inhibition, inhibition of gastric emptying and glucagon secretion (Hay *et al*. 2015). Endogenous amylin and its analogue and receptor agonist, salmon calcitonin (sCT), exert anorexigenic properties by signalling satiation (Lutz *et al*. 1995; Lutz *et al*. 2000; Reidelberger *et al*. 2004; Mack *et al*. 2007; Lutz 2012). Amylin receptors have been identified in various brain areas including area postrema, nucleus of the solitary tract and dorsal raphe among others (Sexton *et al*. 1994). In fact, amylin and sCT express their anorexigenic effects through central mechanisms involving the area postrema and the nucleus of the solitary tract (Lutz *et al*. 2001a; Potes & Lutz 2010; Braegger *et al*. 2014). Recent studies show that amylin receptors in the NAc, VTA and LDTg (Baisley & Baldo 2014; Mietlicki‐Baase *et al*. 2015a; Reiner *et al*. 2017) and more specifically amylin receptors on ventral tegmental dopaminergic neurons mediate the effect of sCT on the control of energy balance (Mietlicki‐Baase *et al*. 2013; Mietlicki‐Baase *et al*. 2015b). Thus, amylin's action on the mesolimbic dopamine system suggests a potential role that extends beyond regulation of natural rewards, and tenuous evidence supports a possible amylinergic mediation of artificial rewards, such as alcohol. Therefore, in the present study, we investigated the effects of sCT on alcohol‐mediated behaviours in mice, namely, locomotor stimulation and accumbal dopamine release as well as the expression of alcohol's rewarding properties and reward‐dependent memory consolidation in the alcohol‐induced conditioned place preference (CPP) paradigm. Moreover, the ability of sCT to influence alcohol intake in low and high alcohol‐consuming rats was explored. Finally, we evaluated the involvement of stress, alcohol metabolism and caloric content as possible mechanisms through which sCT influences alcohol‐induced reward. We hence investigated the effects of sCT on plasma corticosterone levels, blood alcohol concentration and peanut butter consumption, respectively.

## Materials and Methods

For detailed protocol description, see Supporting Information.

### Animals

For the locomotor activity, CPP, *in vivo* microdialysis, peanut butter intake, blood alcohol concentration and corticosterone analysis experiments, adult postpubertal age‐matched male NMRI mice (8–12 weeks old and 25–30 g body weight; Charles River, Susfeldt, Germany) were used. The mice were group housed, fed *ad libitum* and maintained at a 12/12 hour light/dark cycle and at 20°C with 50 percent humidity. Mice were used for the present experiments, because we have extensive experience working with mice and have previously obtained robust locomotor stimulation, CPP and accumbal dopamine release in response to alcohol and other addictive drugs (Vallof *et al*. 2016c).

For the intermittent access, 20 percent alcohol two‐bottle‐choice drinking paradigm, adult postpubertal age‐matched male outbred RccHan Wistar rats (Envigo, Huntingdon, Cambridgeshire, UK) were used. The rats, for the whole duration of this experiment, were maintained on a 12‐hour reversed light/dark cycle (lights off at 8 am) and were housed individually in high Macrolon III cages. They were maintained in rooms with 20°C and 50 percent humidity and fed *ad libitum*. Outbred Rcc Han Wistar rats were selected for the intermittent access paradigm because they display a voluntary high and stable alcohol intake, causing pharmacologically relevant blood alcohol concentrations in the intermittent access model (Vallof *et al*. 2016a).

All experiments were approved by the Swedish Ethical Committee on Animal Research in Gothenburg. Each experiment used an independent set of animals.

### Drugs

Salmon calcitonin (Tocris Bioscience, Bristol, UK) was diluted in vehicle (0.9 percent sodium chloride solution) and administered intraperitoneally (IP) at the doses of 1 or 5 μg/kg always 30 minutes prior to alcohol injection. Alcohol (96 percent, VWR International AB, Stockholm, Sweden) was diluted in vehicle (0.9 percent sodium chloride solution) to 15 percent v/v and was administered at a dose of 1.75 g/kg, IP.

### Locomotor activity

Locomotor activity tests were conducted to investigate the effects of two different doses of sCT (1 or 5 μg/kg, IP) on *per se* locomotor activity in mice and the effects of a high (5 μg/kg, IP) or a low (1 μg/kg, IP) sCT dose on alcohol‐induced locomotor stimulation in mice. For protocol description, see Supporting Information.

Briefly, mice were allowed to habituate to the activity boxes for 60 minutes, and sCT or an equal volume of vehicle (saline solution, IP) was administered 30 minutes prior to alcohol (1.75 g/kg, IP) or vehicle injection. The subsequent 60‐minute cumulative locomotor activity was registered.

### *In vivo* microdialysis and dopamine release measurements

For the measurements of accumbal dopamine release, the mice were implanted with a microdialysis custom‐made probe (Blomqvist *et al*. 1993) positioned in NAc shell (coordinates relative to bregma of 1.5 mm AP, ±0.6 ML and 4.7 mm DV were used (Paxinos & Watson 1998)), after surgical procedures that have been previously described in detail (Jerlhag *et al*. 2009). For protocol description, see Supporting Information.

After 1 hour of habituation to the microdialysis set‐up, perfusion samples were collected in 20‐minute intervals during the entire experimental protocol (from −40 to 260 minutes). The baseline dopamine level was defined as the average of three consecutive samples (−40 to 0 minutes) before the first alcohol (1.75 g/kg, IP) or vehicle (saline solution, IP) challenge (time 0). The dopamine release was determined as the percent increase from baseline.

In the first experiment, an initial alcohol challenge was given to establish that the mice respond with an accumbal dopamine release to alcohol compared with vehicle treatment. Seven consecutive 20‐minute samples were collected after this initial challenge. At 150 minutes, sCT (5 μg/kg, IP) or an equal volume of vehicle (saline solution, IP) was administered. Thirty minutes later, vehicle (saline solution, IP) or alcohol (1.75 g/kg, IP) was administered (180 minutes). Thereafter, four additional samples were collected (experiment terminated at 260 minutes). Collectively, the following three treatment groups were created: alcohol–vehicle–alcohol (Alc‐Veh‐Alc), alcohol–sCT–alcohol (Alc‐sCT‐Alc) and vehicle–sCT–vehicle (Veh‐sCT‐Veh).

In the second microdialysis experiment, the effects of sCT on the initial alcohol‐induced accumbal dopamine release were investigated in animals that received only a single alcohol injection, in order to identify the ability of sCT to affect the initial alcohol‐induced accumbal dopamine release. Firstly, sCT (5 μg/kg, IP) or an equal volume of vehicle (saline solution, IP) was administered after the collection of the three baseline samples at 10 minutes, and 30 minutes later, an alcohol injection (1.75 g/kg, IP) was administered (40 minutes). Thereafter, nine additional samples were collected (experiment terminated at 220 minutes). Collectively, the following two treatment groups were created: vehicle–alcohol (Veh‐Alc) and sCT–alcohol (sCT‐Alc).

### Verification of probe placement

**Figure 1:**
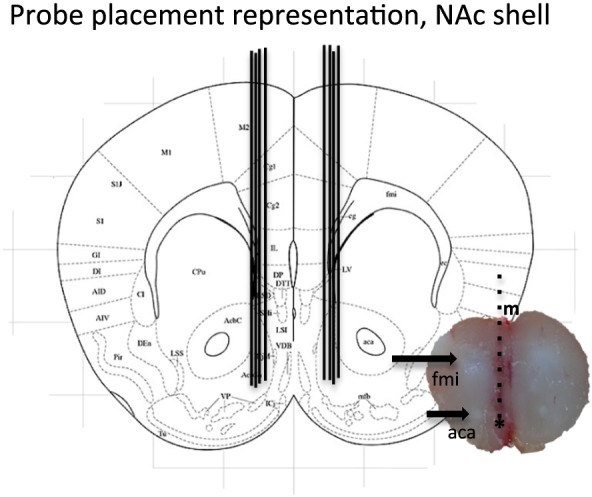
Representative brain slice and schematic representation of probe placement in nucleus accumbens (NAc) shell. A coronal mouse brain section showing eight representative probe placements (illustrated by vertical lines) in the NAc shell. One brain slice shows a representative probe placement in the NAc shell. The asterisk shows the targeted area; vertical lines represent the probe; m, midline; fmi, anterior forceps of corpus callosum; aca, anterior commissure

Following each microdialysis experiment, the probes' location in the brain (NAc shell) was verified. For protocol description, see Supporting Information. Figure 1 shows a schematic representation of placements within NAc shell as well as one representative brain slice verification. Mice with misplaced probe placements were excluded from the statistical analysis.

### Conditioned place preference

Two distinct CPP tests were performed in mice as previously described (Jerlhag *et al*. 2009; Vallof *et al*. 2016a).

The first CPP test was conducted to evaluate the effect of sCT on the rewarding properties of alcohol. In these experiments, sCT or vehicle was administered on the preconditioning days prior to alcohol injections, in order to define the ability of sCT to affect the rewarding properties of alcohol. This experiment allows us to investigate the ability of sCT to prevent the reward induced by acute alcohol, reflecting the expression of CPP. sCT (5 μg/kg, IP) or vehicle (saline solution, IP) was administered 30 minutes prior to alcohol or vehicle (saline solution, IP) administration on each of the four conditioning days, creating two treatment groups of Veh‐Alc and sCT‐Alc.

The second experiment was conducted in a separate mice group and was designed to investigate the effect of sCT on memory consolidation of alcohol reward. In these experiments, sCT or vehicle was administered on the last post‐conditioning day only, whereas alcohol had been administered daily throughout the conditioning period, allowing the mice to establish a place preference for the least preferred compartment. This design tests the ability of sCT to influence the reward‐dependent memory consolidation. At post‐conditioning, mice received sCT (5 μg/kg, IP) or an equal volume of vehicle solution (saline solution, IP) and 30 minutes later placed on the midline between the two compartments with free access to both compartments for 20 minutes (creating the following treatment groups; alcohol–vehicle and alcohol–sCT). For detailed protocol description, see Supporting Information.

### Intermittent access 20 percent alcohol two‐bottle‐choice drinking paradigm

This experiment was conducted to evaluate the effect of sCT (5 μg/kg, IP) on alcohol intake in rats exposed to 12 weeks of alcohol in the intermittent access model.

The rats were given free access to one bottle of 20 percent alcohol and one bottle of water during three 24‐hour sessions per week (Mondays, Wednesdays and Fridays). The rats had unlimited access to two bottles of water between the alcohol‐access periods. All bottles were weighed at 24 hours after the fluids were placed to the rat cages. The body weight of each rat was measured daily prior to bottle presentation, to allow for calculating the grams of alcohol intake per kilogram of body weight (g/kg). The preference for alcohol over water (the ratio of alcohol to total fluid intake) was calculated at all timepoints. In addition, water and food intake was measured. The alcohol intake experiment was conducted after a period of 12 weeks of intermittent access to alcohol.

In this experiment, two separate groups of rats, low and high alcohol consuming, were administered a single injection of sCT (5 μg/kg, IP) or vehicle solution (saline solution, IP) on an alcohol‐drinking day (Monday or Wednesday) in a way that all rats alternately received both sCT and vehicle injections in a balanced design. There was 1 day between each injection (water‐drinking days, Tuesday). After treatment and when returned to their cages, the rats were presented with one bottle of 20 percent alcohol and one bottle of water. Alcohol, water and food intake, as well as alcohol preference measurements, were registered 1 and 24 hours following alcohol presentation. Rat body weight was measured only at the 24‐hour timepoint.

### Peanut butter intake experiments

All mice were allowed to familiarize the taste of peanut butter for 1 week before the test. Mice were transferred to novel, empty cages, and two doses of sCT (1 or 5 μg/kg, IP) or vehicle (saline solution, IP) were administered 30 minutes prior to peanut butter exposure. Peanut butter consumption was measured at the timepoint of 1 hour.

### Blood alcohol concentration

Mice were injected with sCT (5 μg/kg, IP) or an equal volume of vehicle solution (saline solution, IP), and 30 minutes later, all animals were injected with alcohol (1.75 g/kg, IP). The animals were decapitated 20 minutes later, and trunk blood was collected in microtubes (Vacuette, Greiner Bio‐one, Florence, Italy). The analysis of the blood alcohol concentration was outsourced to Sahlghrenska University Hospital (Gothenburg, Sweden; study agreement BML‐NEURO).

### Serum levels of corticosterone

Mice were injected with sCT (5 μg/kg, IP) or an equal volume of vehicle solution (saline solution, IP), and 30 minutes later, capillary blood from the tail was collected in microvettes (Sarstedt, Helsingborg, Sweden). The blood was centrifuged (5 minutes, 10 000 *g*), and corticosterone was thereafter measured in serum with an Enzo Corticosterone enzyme‐linked immunosorbent assay (ELISA) kit (AH Diagnostic, Stockholm, Sweden).

### Statistical analysis

The first experiment of locomotor activity was analysed with a one‐way ANOVA. The further alcohol‐induced locomotor activity experiments were analysed with a two‐way ANOVA test followed by Tukey's *post hoc* test for multiple comparison between treatments. Accumbal dopamine release analyses were performed using a two‐way repeated measures ANOVA followed by Bonferroni *post hoc* test for the comparison between different treatments at given timepoints. CPP, blood alcohol concentration and plasma corticosterone levels data were assessed with an unpaired *t*‐test. The data from the intermittent alcohol access experiment were analysed using a paired *t*‐test. Peanut butter intake was analysed with a one‐way ANOVA followed by a Bonferroni *post hoc* test. Data are presented as mean ± SEM. A probability value of *P* < 0.05 was considered as statistically significant.

## Results

### sCT attenuates alcohol‐induced locomotor stimulation and accumbal dopamine release following a single or repeated alcohol injections in mice

The dose response study showed that there was no effect of sCT (1 or 5 μg/kg, IP) on locomotor activity *per se* in mice when compared with vehicle treatment (*F*(1, 21) = 0.25, *P* = 0.7829); (vehicle: 394 ± 172 cm/60 minutes, sCT 1 μg/kg: 281 ± 64 cm/60 minutes and sCT 5 μg/kg: 317 ± 79 cm/60 minutes).

**Figure 2:**
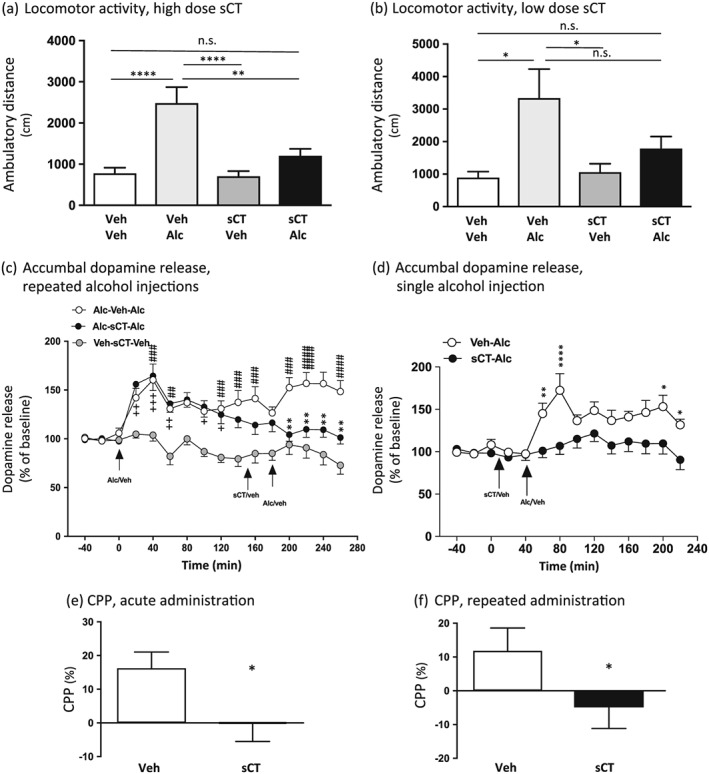
Peripheral injection of salmon calcitonin (sCT) attenuates alcohol‐induced locomotor stimulation, accumbal dopamine release after a single and repeated alcohol injection and the expression of the rewarding properties and reward‐dependent memory consolidation of alcohol‐induced conditioned place preference in mice. (a) Alcohol [Alc, 1.75 g/kg, intraperitoneally (IP)]‐induced locomotor stimulation was blocked by a single peripheral injection of sCT (5 μg/kg, IP), at a dose that does not affect locomotor activity *per se* compared with vehicle (Veh). (Data are presented as mean ± SEM; **P < 0.01, ****P < 0.0001; n.s., non‐significant.) (b) Acute administration of a low dose of sCT (1 μg/kg, IP), a dose without an effect *per se*, had a tendency in reducing alcohol‐induced locomotor stimulation in mice. Indeed, alcohol (1.75 g/kg, IP) did not cause a locomotor stimulation in sCT pretreated mice, but there was not a significant difference between Veh‐Alc‐treated and sCT‐Alc‐treated mice. (Data are presented as mean ± SEM; *P < 0.05; n.s., non‐significant.) (c) An initial alcohol (1.75 g/kg, IP) injection increased accumbal dopamine release for both alcohol‐receiving groups Alc‐Veh‐Alc and Alc‐sCT‐Alc at the timepoints of 20, 40, 60 and 100–160 minutes when compared with Veh‐sCT‐Veh. A second alcohol (1.75 g/kg, IP) injection enhanced accumbal dopamine release in the Alc‐Veh‐Alc group at the timepoints of 200–260 minutes compared with Veh‐sCT‐Veh group. Pre‐treatment with a single peripheral injection of sCT (5 μg/kg, IP), decreased alcohol (1.75 g/kg, IP)‐induced accumbal dopamine release at timepoints 200–260 minutes in the Alc‐sCT‐Alc group compared with Alc‐Veh‐Alc group. (Data are presented as mean ± SEM; ##*P* < 0.01, ###*P* < 0.001, ####*P* < 0.0001 for comparisons between the Alc‐Veh‐Alc and Veh‐sCT‐Veh; +*P* < 0.05, ++*P* < 0.01, +++*P* < 0.001 for comparisons between Alc‐sCT‐Alc and Veh‐sCT‐Veh; **P < 0.01 for comparisons between Alc‐Veh‐Alc and Alc‐sCT‐Alc.) (d) Pre‐treatment with an acute sCT injection (5 μg/kg, IP) blocked the initial accumbal dopamine release caused by a single alcohol (1.75 g/kg, IP) injection in the sCT‐Alc group when compared with the Veh‐Alc, at the timepoints of 60, 80, 200 and 220 minutes. (Data are presented as mean ± SEM; **P < 0.01, ****P < 0.0001, *P < 0.05.) (e) Repeated administration of sCT (5 μg/kg, IP) blocks the rewarding effect of alcohol (1.75 g/kg, IP)‐induced conditioned place preference (CPP). (Data are presented as mean ± SEM, *P < 0.05; data calculated as percent of total time spent in the drug‐paired (i.e. less preferred) compartment during post‐conditioning and preconditioning sessions.) (f) A single injection of sCT on the post‐conditioning day blocked the reinforcing memory consolidation of alcohol (1.75 g/kg, IP)‐induced CPP. (Data are presented as mean ± SEM, *P < 0.05.)

A high dose of sCT attenuated alcohol‐induced locomotor stimulation. Indeed, an effect of alcohol treatment (*F*(1, 49) = 23.35, *P* < 0.0001) and of sCT treatment (*F*(1, 49) = 8.75, *P* = 0.0048) was found on locomotor activity after sCT (5 μg/kg, IP) and alcohol (1.75 g/kg, IP) administration. There was an effect of alcohol × sCT treatment interaction (*F*(1, 49) = 6.97, *P* = 0.0111; *n* = 14 for vehicle–vehicle and sCT–vehicle, *n* = 13 for Veh‐Alc and *n* = 12 for sCT‐Alc group) on locomotor activity. Further *post hoc* analysis showed that alcohol significantly increased locomotor activity in comparison with vehicle treatment (*P* < 0.0001; Fig. 2a. This effect was significantly attenuated by a single injection of sCT prior to alcohol administration (*P* < 0.01) at a dose with no effect *per se* on locomotor stimulation as shown by comparison with the vehicle group (*P* > 0.05). There was no significant difference between the vehicle group and the sCT–alcohol group (*P* > 0.05).

On the contrary, a low dose of sCT reduced but did not completely attenuate alcohol‐induced locomotor stimulation. There was an effect of alcohol treatment (*F*(1, 23) = 9.39, *P* = 0.0055) on locomotor activity following a low dose of sCT (1 μg/kg, IP) and alcohol (1.75 g/kg, IP) administration, but no effect of sCT treatment (*F*(1, 23) = 2.02, *P* = 0.1680) or alcohol × sCT treatment interaction (*F*(1, 23) = 2.36, *P* = 0.1384; *n* = 8 per group) was found on locomotor activity (Fig. 2b). Further *post hoc* analysis showed that alcohol treatment increased locomotor activity in vehicle pretreated mice compared with vehicle treatment (*P* < 0.05). Albeit alcohol did not increase locomotor activity in mice pretreated with sCT (*P* > 0.05), there was no significant difference between vehicle–alcohol and sCT–alcohol treatment (*P* > 0.05). The selected dose of 1 μg/kg had no effect *per se* on locomotor activity compared with vehicle treatment (*P* > 0.05).

As shown in Figure 2c, the accumbal dopamine release data showed an overall effect of treatment (*F*(2, 384) = 103, *P* < 0.0001), time (*F*(15, 192) = 5.953, *P* < 0.0001) and treatment × time interaction (*F*(30, 384) = 3.065, *P* < 0.0001; *n* = 13 per group).

In the first part of the experiment, the responsiveness to alcohol (1.75 g/kg) *per se* was investigated (alcohol injection at timepoint 0 minutes). This initial injection of alcohol caused a significant increase in accumbal dopamine release compared with vehicle treatment (Veh‐sCT‐Veh) in both groups that received alcohol (Alc‐Veh‐Alc and Alc‐sCT‐Alc). Specifically, in the Alc‐Veh‐Alc group, alcohol significantly increased accumbal dopamine at timepoints 40 (*P* < 0.001), 60 (*P* < 0.01), 120 (*P* < 0.01), 140–160 (*P* < 0.001) and 180 minutes (*P* < 0.05). Moreover, alcohol increased dopamine in NAc at timepoints 20 (*P* < 0.01), 40 (*P* < 0.001), 60 (*P* < 0.01) and 100–120 minutes (*P* < 0.05) in the Alc‐sCT‐Alc group. The subsequent part of the experiment aimed at investigating the ability of sCT to affect alcohol‐induced dopamine release. Administration of sCT (5 μg/kg, IP at 150 minutes) 30 minutes prior to the second alcohol injection (1.75 g/kg, IP at 180 minutes) significantly decreased the alcohol‐induced accumbal dopamine release (Alc‐sCT‐Alc) compared with vehicle pre‐treatment (Alc‐Veh‐Alc) at the timepoints 200–260 (*P* < 0.01).

The second accumbal dopamine release experiment (Fig. 2d) revealed an overall effect of treatment (*F*(1, 126) = 54.56, *P* < 0.0001), time (*F*(13, 126) = 4.722, *P* < 0.0001) and time × treatment interaction (*F*(13, 126) = 2.634, *P* = 0.0028; *n* = 10 per group). *Post hoc* analysis revealed that administration of sCT (5 μg/kg, IP at 10 minutes) 30 minutes prior to a single alcohol injection (1.75 g/kg, IP at 40 minutes) significantly decreased alcohol‐induced accumbal dopamine release (sCT‐Alc) compared with vehicle pre‐treatment (Veh‐Alc) at the timepoints 60 (*P* < 0.01), 80 (*P* < 0.0001), 200 (*P* < 0.05) and 220 (P < 0.05) minutes.

### sCT attenuates the expression of alcohol‐induced CPP in mice

The first CPP experiment revealed that sCT significantly attenuated the rewarding properties of alcohol, i.e. expression of CPP (*P* = 0.0420; *n* = 8 per group; Fig. 2e). sCT had no effect on CPP in this experimental set‐up as resulted by a separate control experiment [−2 ± 1 percent for vehicle–vehicle (*n* = 8) and −2 ± 2 percent for sCT–vehicle (*n* = 7); *P* = 0.8471]. As shown in Figure 2f**,** a single injection of sCT (5 μg/kg, IP) on the post‐conditioning day significantly decreased memory consolidation of alcohol reward in the CPP test (*P* = 0.0453, *n* = 8 per group). Finally, acute administration of sCT on the post‐conditioning day did not affect CPP *per se* in mice as resulted from a separate experiment [1 ± 5 percent for vehicle–vehicle (*n* = 8) and 5 ± 4 percent for vehicle–sCT (*n* = 7); *P* = 0.5996].

### sCT decreases alcohol and food intake in low alcohol‐consuming rats

**Figure 3:**
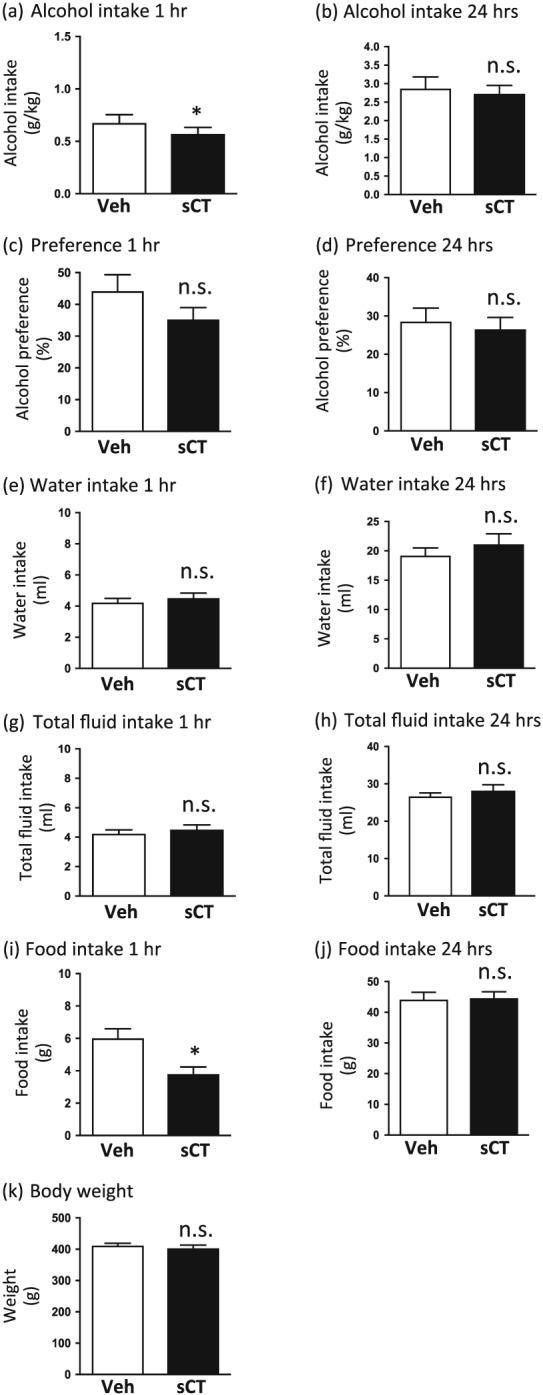
Salmon calcitonin (sCT) administration decreases alcohol and food intake in low alcohol‐consuming rats. (a) A single sCT injection [5 μg/kg, intraperitoneally (IP)] decreased alcohol intake in low alcohol‐consuming rats in the intermittent access 20 percent alcohol two‐bottle‐choice drinking paradigm at the timepoint of 1 hour compared with vehicle (Veh). (b) sCT administration did not have an effect on alcohol intake at the timepoint of 24 hours. There was no effect of sCT on alcohol preference in either timepoint of (c) 1 hour or (d) 24 hours. sCT did not alter water intake at either measured timepoint of (e) 1 hour or (f) 24 hours. sCT did not alter the total fluid intake at either timepoint of (g) 1 hour or (h) 24 hours. A single sCT injection decreased food intake at the timepoint of (i) 1 hour but had no effect at the timepoint of (j) 24 hours. sCT did not alter 24‐hour rat body weight in the low alcohol‐consuming group (k). (Data are presented as mean ± SEM; *P < 0.05; n.s., non‐significant.)

A single injection of sCT (5 μg/kg, IP) significantly reduced alcohol intake in low alcohol‐consuming rats (mean baseline alcohol consumption: 2.31 g/kg) compared with vehicle injection, as shown in Figure 3a, at the timepoint of 1 hour (*P* = 0.0177, *n* = 17). Alcohol intake scores for the 24‐hour timepoint were not significantly different between the two groups (*P* = 0.4477; Fig. 3b). Overall, the sCT‐treated group showed a decrease of 15 percent in alcohol consumption (mean between the treatment groups: 0.6741–0.5712 g/kg) for the timepoint of 1 hour and a decrease of 5 percent (mean baseline consumption between the treatment groups: 2.866–2.726 g/kg) for the timepoint of 24 hours.

sCT administration did not alter alcohol preference in either measured timepoint of 1 hour (*P* = 0.0917; Fig. 3c) or 24 hours (*P* = 0.2401; Fig. 3d). sCT did not alter water intake at any measured timepoint of 1 hour (*P* = 0.1255; Fig. 3e) or 24 hours (*P* = 0.0783; Fig. 3f). There was no effect of sCT on the total fluid consumption in the rats at any measured timepoint of 1 or 24 hours (*P* = 0.3976 and *P* = 0.182, respectively; Fig. 3g & h).

Acute administration of sCT significantly decreased food intake at the timepoint of 1 hour after administration compared with vehicle administration (*P* = 0.0075) as presented in Figure 3i. Food intake scores at the timepoint of 24 hours after administration did not significantly differ between the two conditions (*P* = 0.8946; Fig. 3j). sCT administration did not have an effect on rat weight (*P* = 0.1980) in low alcohol‐consuming rats (Fig. 3k).

### sCT robustly decreases alcohol, food intake and rat body weight in high alcohol‐consuming rats

**Figure 4:**
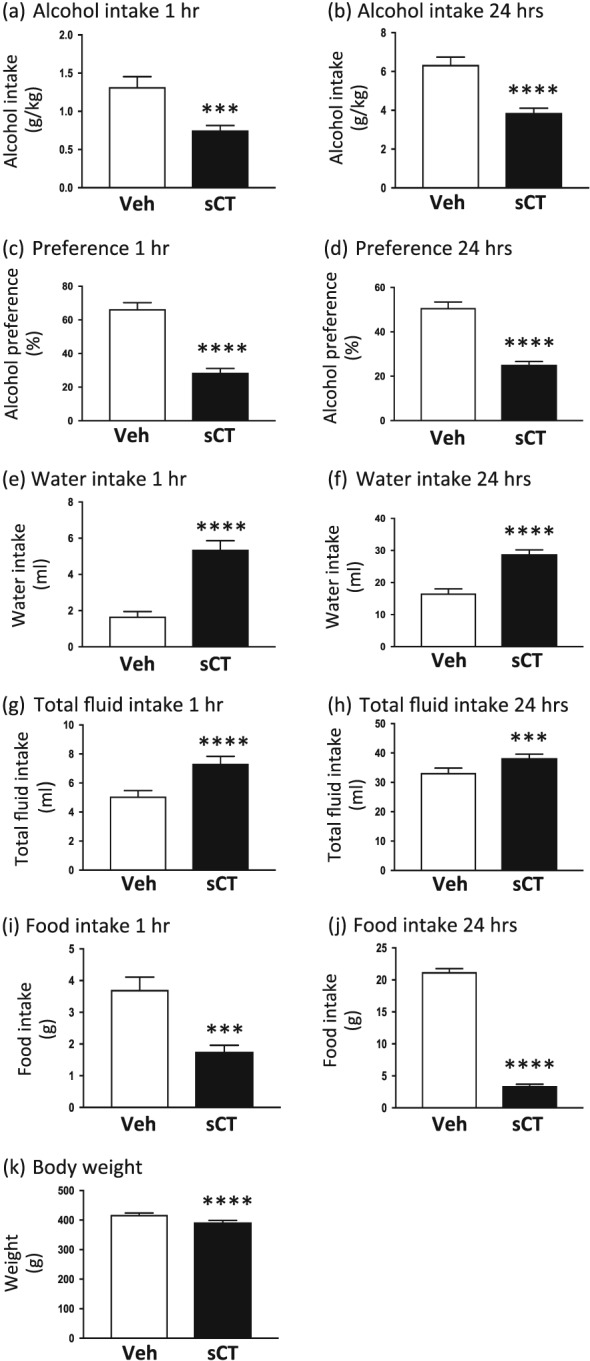
Salmon calcitonin (sCT) administration decreases alcohol and food intake, as well as body weight in high alcohol‐consuming rats. Acute sCT administration [5 μg/kg, intraperitoneally (IP)] reduced alcohol intake in high alcohol‐consuming rats in the intermittent access 20 percent alcohol two‐bottle‐choice drinking paradigm at the timepoint of (a) 1 hour and (b) 24 hours compared with vehicle (Veh). sCT pre‐treatment decreased preference for alcohol at the timepoints of both (c) 1 hour and (d) 24 hours. sCT increased (e) 1‐hour and (f) 24‐hour water intake and similarly increased total fluid intake at the timepoints of (g) 1 hour and (h) 24 hours. A single sCT injection decreased food intake at the timepoint of (i) 1 hour and (j) 24 hours. sCT administration decreased body weight at 24 hours in the high alcohol‐consuming group (k). (Data are presented as mean ± SEM; ****P < 0.0001, ***P < 0.001.)

A single injection of sCT (5 μg/kg, IP) significantly reduced alcohol intake in high alcohol‐consuming rats (mean baseline alcohol consumption: 6.14 g/kg) compared with vehicle injection, as shown in Figure 4a, at the timepoint of 1 hour (*P* = 0.001, *n* = 25). Alcohol intake scores for the 24‐hour timepoint showed that sCT significantly reduced alcohol intake when compared with the vehicle group (*P* = 0.0001; Fig. 4b) in these high alcohol‐consuming rats. Collectively, the sCT‐treated group showed a 43 percent decrease in alcohol intake (mean consumption between the treatment groups: 1.317–0.7484 g/kg) for the timepoint of 1 hour and 39 percent decrease at the 24‐hour timepoint (mean consumption between the treatment groups: 6.343–3.862 g/kg).

Acute sCT administration decreased alcohol preference at the timepoint of 1 hour (*P* < 0.0001; Fig. 4c) and 24 hours (*P* < 0.0001; Fig. 4d). sCT significantly increased water intake at timepoints of both 1 hour (*P* < 0.0001; Fig. 4e) and 24 hours (*P* < 0.0001; Fig. 4f) compared with the vehicle‐treated group. Moreover, total fluid intake was significantly increased after sCT administration at the timepoint of 1 hour (*P* < 0.0001; Fig. 4g) and 24 hours (*P* = 0.001; Fig. 4h) when compared with the vehicle group.

Acute administration of sCT significantly reduced food intake at the 1‐hour (*P* = 0.0001; Fig. 4i) and 24‐hour (*P* < 0.0001; Fig. 4j) timepoints compared with vehicle in the high alcohol‐consuming rats. Moreover, a single sCT injection significantly decreased the 24‐hour values of rat body weight (*P* < 0.0001; Fig. 4k).

### sCT does not alter peanut butter intake in mice

**Figure 5:**
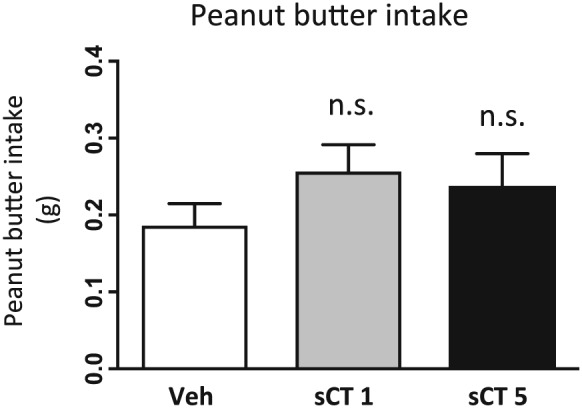
Salmon calcitonin (sCT) administration does not affect intake of palatable food in satiated mice. Acute sCT administration in two different doses [1 and 5 μg/kg, intraperitoneally (IP)] did not change peanut butter consumption compared with vehicle (Veh) administration. (Data are presented as mean ± SEM; n.s., non‐significant.)

sCT administration in two doses (low: 1 μg/kg, IP and high: 5 μg/kg, IP) did not alter peanut butter intake compared with vehicle as shown in Figure 5, at the timepoint of 1 hour (*F*(2, 29) = 0.9771, *P* = 0.3884; *n* = 10 for the vehicle and *n* = 11 for both sCT groups).

### sCT does not alter blood alcohol concentration and corticosterone levels

**Figure 6:**
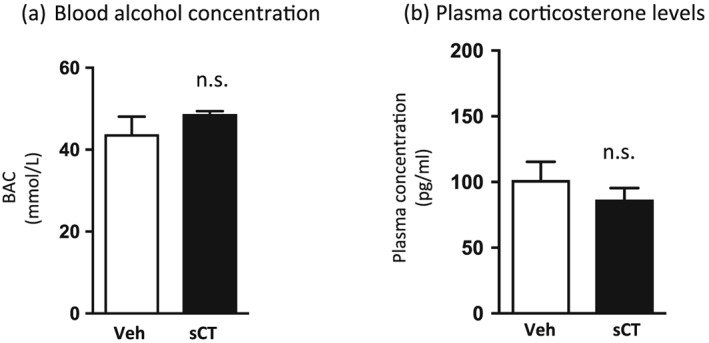
Salmon calcitonin (sCT) does not affect blood alcohol concentration and plasma corticosterone levels in mice. A single sCT injection [5 μg/kg, intraperitoneally (IP)] did not alter (a) the blood alcohol (1.75 g/kg, IP) concentration (BAC) and (b) the plasma levels of corticosterone compared with vehicle (Veh) administration. (Data are presented as mean ± SEM; n.s., non‐significant.)

Figure 6a shows the blood alcohol concentration levels between vehicle (saline solution, IP) and sCT (5 μg/kg, IP)‐treated groups. sCT administration did not affect blood alcohol concentration (*P* = 0.2733, *n* = 8 per group).

A single sCT (5 μg/kg, IP) injection did not affect plasma corticosterone levels compared with vehicle injection, as presented in Figure 6b (*P* = 0.3549, *n* = 6 in sCT and *n* = 8 in vehicle group).

## Discussion

To the best of our knowledge this is the first study presenting association between amylin receptor signalling and alcohol‐mediated behaviours in rodents. Indeed, we show that sCT, an amylin receptor agonist, attenuates alcohol‐induced locomotor stimulation, accumbal dopamine release, after both a single and a repeated alcohol injection, and the expression of reward‐associated as well as reinforcing memory‐dependent CPP in mice. Moreover, we demonstrate that sCT reduces alcohol and food consumption in low as well as high alcohol‐consuming rats with a more robust effect in the high consumers. Our findings also show that sCT alters neither blood alcohol concentration nor plasma corticosterone levels in mice. Finally, we demonstrate that sCT does not affect peanut butter intake in mice.

The present data show that positive regulation of amylin receptors affects dopaminergic neurotransmission at the level of the mesolimbic dopamine system by preventing alcohol to activate this system. Importantly, the present microdialysis experiments show that sCT decreased alcohol‐induced dopamine release after both a single and two repeated alcohol injections, thus indicating that sCT blocks the initial dopamine release caused by alcohol as well as subsequent dopamine release after a second alcohol injection. In support for a role of amylin in reward regulation are previous data linking dopamine with amylin‐mediated natural rewards, as shown by amylinergic regulation of sexual behaviour in rats through inhibition of dopamine transmission (Clementi *et al*. 1999), as well as by activation of dopamine D2 receptors in neurons of the AP in regard to amylin's satiety effects (Lutz *et al*. 2001b). Moreover, activation of the amylin receptors on dopaminergic neurons of the VTA in rats decreases phasic dopamine in NAc core (Mietlicki‐Baase *et al*. 2013). Another study showing decreased amphetamine‐induced locomotor stimulation in rats after intracerebroventicular sCT administration (Twery *et al*. 1986) supports that amylin signalling activation regulates drug‐induced reward. However, in this study, sCT decreased locomotion and that is in contrast with our results that revealed no effect of sCT on locomotor activity *per se*. Our data revealed that a low dose of sCT (1 μg/kg) did not block but presented a dose‐dependent effect in regard to alcohol‐induced locomotor stimulation. Experiments in rhesus monkeys have showed that administration of lower doses of sCT, in the range of 0.032 to 1 μg/kg, decreases food intake in a dose–response manner (Bello, Kemm, & Moran 2008), suggesting a potential enhanced effect of higher doses of the drug. However, the absence of significant sCT effect in our locomotor activity experiments could potentially be explained by the requirement of higher doses of sCT in order to exert its alcohol antagonizing effects. Although this is the first study demonstrating a direct link between peripheral administration of sCT and decreased alcohol intake in rats, indirect data have showed that the calcitonin gene‐related peptide, a member of the calcitonin family, was detected in lower levels in the hippocampus and frontal cortex of alcohol‐preferring rats compared with non‐preferring (Ehlers *et al*. 1999).

In the present study, we showed that sCT administered on the post‐conditioning day blocked reinforcing memory consolidation in the CPP paradigm in mice. In support for a role of amylin receptor activation in memory processes are the data showing that peripherally administered amylin enhances memory in mice under training conditions in a T‐maze paradigm (Flood & Morley 1992; Flood *et al*. 1995). More recent studies show that long‐term peripheral amylin treatment enriched learning and memory in mouse models of Alzheimer's disease (Zhu *et al*. 2015; Qiu 2017; Zhu *et al*. 2017), suggesting amylin receptors as a drug target for potential treatment of the disease (Qiu 2017). Moreover, a human study showed a positive correlation of plasma amylin and improved cognitive function in elderly population, suggesting a defensive role of amylin receptors against cognitive inclination (Qiu *et al*. 2014). A possible, although speculative, explanation of our results could suggest that amylinergic activation leads to correction of reward‐dependent memory consolidation and diminishes its expression in the CPP paradigm. However, the brain areas and mechanisms involved in amylinergic memory regulation remain to be investigated in detail in further studies.

It could be hypothesized that the noted effects of alcohol‐mediated behaviours could fall beyond the scope of reward. A possible explanation could be that sCT changes alcohol metabolism or causes stress‐related symptoms that could alter alcohol consumption. However, our data show that sCT administration did not affect the levels of alcohol concentration or corticosterone in the blood, thus ruling out the implication of the aforementioned factors. Nausea or taste aversion could also explain the differential response to sCT. It has been shown that sCT does not cause any side effects like nausea, aversion or malaise (Mietlicki‐Baase *et al*. 2013). Moreover, in our experiments, sCT does not affect the preference *per se* in either CPP test, indicating that it does not condition for aversion in mice. Another tentative possibility might be that sCT reduces alcohol intake because of alcohol's caloric content, and indeed, we see that sCT reduces food intake in rats. However, in this study, we did not find an effect of sCT on peanut butter consumption in mice. The lack of effect on a highly caloric food led us to the hypothesis that the remarked effects of sCT on alcohol do not appear to be calorically regulated. On that note, results showing that sCT blocks amphetamine‐induced locomotor stimulation (Twery *et al*. 1986) do not support a role of caloric regulation but rather that of reward regulation. Another explanation for our results could lie in the fact that sCT affects other systems. Indeed, sCT has been proposed to act as an analgesic possibly by causing a CNS increase in beta‐endorphin levels (Franceschini *et al*. 1993), and previous studies have showed that antagonism of the signalling system of another gut–brain peptide, that of ghrelin, alters the levels of endogenous opioids (Engel, Nylander, & Jerlhag 2015); thus, upcoming studies could define other signalling systems involved in amylin‐regulated reward. Another explanation for our results could lie in the binding selectivity of sCT that could affect other receptor pathways. However, this appears less likely because research identifies that sCT binds selectively to calcitonin receptors, the core component of amylin receptors (Barwell *et al*. 2012) and irreversibly and with higher affinity on amylin receptors than amylin (Lutz *et al*. 2000); a selective binding on amylin receptors is also supported by the fact that the doses used in the present do not have an effect *per se* on locomotor activity and CPP in mice. Peripherally, sCT binds to calcitonin receptors on bone osteoclasts (Chesnut et al. 2008; Nicholson et al. 1986) and the kidney (Marx, Woodard, & Aurbach 1972), and it has been used for the treatment of bone metabolic diseases that involve these receptors, for example, osteoporosis (Munoz‐Torres, Alonso, & Raya 2004). It is well established that direct activation of calcitonin receptors by sCT on osteoclasts inhibits bone resorption and activation of renal receptors enhances calcium excretion. Thus, possible effects of the drug's binding to these peripheral receptors cannot be disregarded. However, inhibited bone resorption would not seem to explain the effects of sCT on the alcohol‐induced activation of the mesolimbic dopamine system, i.e. accumbal dopamine release, or the expression of alcohol‐induced CPP, which reflect reward processing (Bardo & Bevins 2000; Boileau *et al*. 2003). Another tentative explanation of our findings could be that enhanced calcium levels after sCT activation of renal calcitonin receptors reflect an increase in brain calcium levels consequently changing calcium influx/efflux in neuronal ion channels. On a similar note, a recent clinical study investigating the effects of acamprosate on alcohol‐dependent individuals attributed the drug's effects to calcium levels and not to a specific CNS target (Spanagel *et al*. 2014). Nevertheless, a number of studies investigating the effects of locally administered sCT on food intake accompanied by immunohistochemistry data have identified central sites of action, especially in areas of the mesolimbic dopamine system (Mietlicki‐Baase *et al*. 2013; Mietlicki‐Baase & Hayes 2014; Reiner *et al*. 2017). Thus, we argue that the present findings do not fall under the scope of calcium levels regulation, but they are a result of sCT's central action. Although there is no robust evidence that sCT crosses the blood–brain barrier effectively, there are data showing that peripheral sCT did not affect food intake in rats with a knocked down calcitonin receptor in the VTA but decreased food intake in control (Mietlicki‐Baase *et al*. 2015b). Moreover, a recent study showed that peripherally administered sCT decreased VTA‐evoked phasic dopamine release in the NAc (Whiting *et al*. 2017), supporting our notion that sCT acts centrally on the mesolimbic dopamine system to regulate dopamine neurotransmission. In line with the present results, we therefore suggest that sCT crosses the blood–brain barrier and reaches areas of the midbrain to regulate alcohol reinforcement. Thus, upcoming studies should explore and define the brain areas involved in the ability of sCT to regulate alcohol‐mediated behaviours. Moreover, in light of the findings that amylin receptor inhibition increases food intake as well as body adiposity in rats (Rushing *et al*. 2001; Reidelberger *et al*. 2004), future experiments exploring the possibility that an amylinergic receptor antagonist increases alcohol intake are warranted.

Studies have presented that amylin receptor signalling in VTA, NAc and LDTg (Baisley & Baldo 2014; Mietlicki‐Baase *et al*. 2015b; Reiner *et al*. 2017) is involved in the control of food intake. Albeit these areas are part of the cholinergic–dopaminergic pathway, which is well established as an important pathway involved in reward‐related behaviours (Soderpalm & Ericson 2013), very little is known about the role of amylin receptor signalling within these areas in regard to artificial reinforcers, such as alcohol. It is now known that other gut peptides like ghrelin, glucagon‐like peptide‐1 and neuromedin U regulate both natural and artificial reward behaviours by acting on receptors located throughout this reward link (Egecioglu *et al*. 2010; Egecioglu *et al*. 2013b; Jerlhag & Engel 2011; Suchankova *et al*. 2013b; Vallof *et al*. 2016c)**.** We therefore hypothesize that amylin receptors in these reward‐related brain areas could act as mediators of reward, potentially devaluating the incentive value of alcohol after their activation; however, local administration in sites of the mesolimbic dopamine pathway will reveal more about the neurobiological mechanisms involved.

In the present study, we show that acute administration of sCT affects alcohol, food and water intake as well as body weight and that this effect is more pronounced in high alcohol‐consuming than low alcohol‐consuming rats. sCT decreased short‐term (1‐hour values) food intake in rats, without altering the rats' weight in this low alcohol‐consuming group. However, sCT decreased both short‐term and long‐term (1‐ and 24‐hour values) food intake in high alcohol‐consuming rats, which was accompanied by a decrease in body weight. The short‐term effect of sCT on food intake in the low alcohol consumers is compatible with other food intake studies, where various doses of sCT suppress food intake in a dose‐dependent manner, showing the most robust effect on the first meal size (Bello *et al*. 2008). Moreover, the present results are in accordance with previous studies that have established an anorexigenic effect of the activation of amylin receptors (Reda *et al*. 2002). The remarked differences in food intake between the two groups show that high alcohol‐consuming rats are more sensitive to the anorexigenic effect of sCT, and possibly due to changes in the brain after long‐term high alcohol exposure. Importantly, we noted a robust decrease in the rat body weight after sCT administration in the high alcohol‐consuming group that was absent in the low alcohol‐consuming one. This is in corroboration with previous studies showing that peripheral sCT decreases 24‐hour body weight in outbred rats (Shah & Donald 1984) and causes decrease of body weight in diet‐induced obese rats (Feigh *et al*. 2011). Supportively, clinical data have showed that amylin analogues like pramlintide reduce body weight in overweight/obese and diabetic (type 2) patients compared with placebo, with the effect being more pronounced in markedly obese patients (Hollander *et al*. 2004). The discrepancy of the data between the two alcohol‐consuming groups strengthens the hypothesis that chronic exposure to high alcohol could possibly alter brain neurocircuits and increase the sensitivity to sCT in regard to body weight regulation. On a similar note, we found that sCT reduces alcohol intake at the first hour after administration, but not 24 hours later in the low alcohol‐consuming group; however, it dramatically decreased the 1‐ and 24‐hour alcohol intake in the high alcohol‐consuming rats. Indeed, the percentage of alcohol change is more robust in high compared with low alcohol‐consuming rats. In support for this robust effect in high consumers are the data showing that sCT reduces alcohol preference in these rats but not in low consumers. Moreover, sCT did not affect water and total fluid intake in the low alcohol‐consuming group, whereas it robustly increased it in the high alcohol‐consuming one. This increase in water intake could be attributed to the compensation of drinking behaviour in the high alcohol‐consuming group, as a consequence of the robust alcohol intake decrease. The absence of long‐term effect of sCT on alcohol intake in the first experiment could be attributed to the half‐life of the drug, which has been shown to be rapidly absorbed with peak concentration in blood plasma within 30–60 minutes after subcutaneous administration (Sinko et al.). Nevertheless, the effect of sCT is very pronounced in the 24‐hour values in the high alcohol‐consuming group, indicating different sensitivity of the two consuming groups to sCT in regard to alcohol intake regulation. In accordance are studies showing that central administration of neuromedin U dose dependently decreases alcohol intake in high but not in low alcohol‐consuming rats (Vallof *et al*. 2016b). Moreover, administration of a ghrelin antagonist reduced alcohol intake more robustly in rats voluntarily exposed to alcohol for 5 months instead of 2 (Landgren *et al*. 2012). As previously mentioned, a tentative explanation could be that chronic exposure to high alcohol consumption can lead to alterations in the brain circuits involved in sCT alcohol reward regulation. Indeed, alcohol‐preferring and high alcohol‐drinking rats were found to have fewer calcitonin gene‐related peptide receptor‐binding sites in forebrain regions compared with non‐preferring and low alcohol‐drinking rats, respectively (Hwang *et al*. 1995). With focus on the present data, a speculative explanation could lie on the ability of sCT to more robustly decrease alcohol intake as a result of altered sensitivity in the brain. Nevertheless, gene expression studies and other molecular approaches would reveal more about these differences in the brain and are warranted in the future.

Interestingly, sCT administered in two different doses did not alter peanut butter intake in satiated mice. Our results are contradicting to previous studies showing that sCT decreases lever pressing for palatable reward (Morley *et al*. 1997) and palatable food intake in mice (Eiden *et al*. 2002). However, either these studies used high doses of amylin in the range of 100 to 200 μg/kg in an operant paradigm (Morley *et al*. 1997), or they were conducted in mice resistant to leptin after being scheduled on chocolate as a highly caloric substitute to chow for more than 40 days (Eiden *et al*. 2002). Another difference is that our experiments were conducted in novel cages; thus, novelty could be considered as a factor potentially influencing our data. It has been shown that palatable food consumption is inversely regulated in different energy statuses, as proved by studies with other gut–brain peptides. Given that the mice used in our experiments were satiated, our results are in accordance with studies showing that ghrelin does not increase palatable food intake in *ad libitum* fed mice, but it does so in fasted mice (Alen *et al*. 2013). This is corroborated by our previous data presenting that ghrelin does not increase peanut butter intake in satiated mice (Kalafateli *et al*. 2017) and that its administration in rats scheduled for palatable feeding decreases high‐fat‐diet consumption and enhances normal chow intake (Schéle *et al*. 2016; Bake, Hellgren, & Dickson 2017). It is therefore possible that the amylinergic mechanisms regulating food reward do not coincide with the ones mediating substance reinforcement.

Collectively, we show for the first time that a single peripheral injection of sCT attenuates alcohol‐mediated behaviours in rodents by decreasing alcohol's ability to activate the mesolimbic dopamine system. Importantly, amylin analogues like pramlintide for the treatment of diabetes and sCT products for the treatment of osteoporosis and Paget's disease are already commercially available. Providing that research on neurochemical mechanisms through which alcohol activates the mesolimbic dopamine reward link has led to identification of novel treatment targets (Edwards *et al*. 2011; Engel & Jerlhag 2014) and in combination with our preclinical data, the aforementioned or similar agents could tentatively be used as potential treatment of alcohol dependence as well as other addiction disorders.

## Supporting information


**Data S1.** Supporting information
