# Supplementary material for: Activation of amylin receptors attenuates alcohol‐mediated behaviours in rodents
Source: Addict Biol. 2018 Feb 6;24(3):388–402. doi: 10.1111/adb.12603 (PMC6585842; doi:10.1111/adb.12603)
Supplement: Supplementary file 1 — Data S1. Supporting information [file ADB-24-388-s001.pdf]

SUPPLEMENTARY INFORMATION

The following contains detailed protocol information of the Material and Methods section.

Drugs

The dose of 5 µg/kg was selected as the most extensively used in previous studies (Braegger *et al*, 2014; Lutz *et al*, 2000; Mietlicki-Baase *et al*, 2013; Pecile *et al*, 1987) and as a dose with no effect on locomotor activity *per se* in our pilot experiment (vehicle: 394±172 cm/60 minutes and sCT 5 µg/kg: 317±79 cm/60 minutes). The lower dose of 1 µg/kg was selected based on our pilot experiment data showing that this dose had no effect *per se* on locomotor activity (vehicle: 394±172 cm/60 minutes and sCT 1 µg/kg: 281±64 cm/60 minutes). Providing that 5 µg/kg, but not 1 µg/kg, attenuated locomotor stimulation induced by alcohol, this higher dose was used in all following studies. The dose of alcohol was selected as it has been shown to cause a robust locotomor stimulation, accumbal dopamine release and expresion of conditioned place preference in mice (Egecioglu *et al*, 2013; Jerlhag *et al*, 2006).

Locomotor activity experiments

Locomotor activity was registered in six sound attenuated, ventilated and dim lit locomotor boxes (420 x 420 x 200 mm; Open Field Activity System; Med Associates Inc;

Georgia, Vermont, USA). In this system, 15 x 15 infrared beams at the bottom of the floor allow a computer-based system to register the distance travelled (cm per 5 minutes) of each mouse during 60 minutes. Prior to drug administrations the mice were allowed to habituate to the locomotor activity box for 60 minutes.

In the first experiment, the effects of sCT (5 µg/kg, IP) on locomotor activity were investigated. sCT or vehicle (saline solution, IP) was administered and the subsequent 60-minute cumulative locomotor activity was registered.

In the second experiment, the effects of sCT (5 µg/kg, IP) on alcohol-induced (1.75 g/kg, IP) locomotor stimulation were investigated. sCT or an equal volume of vehicle (saline solution, IP) was administered 30 minutes prior to alcohol or vehicle injection. This time interval was selected after exploratory studies showing that the drug effect took place 30 minutes following administration. Alcohol was administered subsequently and locomotor activity registration started 5 minutes following the last injection. Each mouse was subjected to only one experimental trial and received one of the following treatment combinations in a 2 x 2 factorial between subjects design: vehicle-vehicle, sCT-vehicle, vehicle-alcohol, sCT-alcohol.

In the third experiment, the effects of a lower dose of sCT (1 µg/kg, IP) on alcohol-induced (1.75 g/kg, IP) locomotor stimulation were investigated. sCT or an equal volume of vehicle (saline solution, IP) was administered 30 minutes prior to alcohol or vehicle injection. Alcohol was administered subsequently and after 5 minutes the locomotor activity registration started. Each mouse was subjected to only one experimental trial

and received one of the following treatment combinations in a 2 x 2 factorial between subjects design: vehicle-vehicle, sCT-vehicle, vehicle-alcohol, sCT-alcohol.

***In vivo* microdialysis and dopamine release measurements**

The microdialysis experiment, in freely moving mice, was designed to establish an initial response to alcohol as well as to explore the effect of sCT on alcohol-induced dopamine release.

The mice were anesthetized with isoflurane (Isoflurane Baxter; Univentor 400 Anaesthesia Unit, Univentor Ltd., Zejtun, Malta), placed in a stereotaxic frame (David Kopf Instruments; Tujunga, CA, USA) and kept on a heating pad to prevent hypothermia. The skull bone was exposed and one hole for the probe and one for the anchoring screw were drilled. Two drops of Xylocaine adrenaline (5 µg/ml; Pfizer Inic; New York, USA) was used as local anesthetic. The probe was randomly alternated to either the left or the right side of the brain in a balanced setup. The coordinates relative to bregma of 1.5 mm AP, ±0.6 ML and 4.7 mm DV were used (Paxinos and Watson, 1998), aiming at NAc shell. The shell was selected, as there is robust evidence that drugs of abuse, including alcohol increase dopamine release selectively in NAc shell, but not in the core (Bassareo and Di Chiara, 1999; Cadoni *et al*, 2000). All probes were surgically implanted two days prior to the experiment. After surgery, the mice were injected with carprofen (5 mg/kg subcutaneous, Rimadyl®; Astra Zeneca, Gothenburg, Sweden) to relieve pain and were kept in individual cages (Macrolon III).

During the microdialysis experiment, the probe was connected to a microperfusion pump (U-864 Syringe Pump; AgnThós AB) and perfused with Ringer solution at a rate of 1.5 µl/minute.

Dopamine was separated and quantified using two different high-performance liquid chromatography apparatuses with electro chemical detection as described previously (Clarke *et al*, 2014). In brief, a pump (UltiMate 3000 Pump; Thermo Scientific, Darmstadt, Germany), an ion exchange column (Nucleosil SA, 2.0 x 150 mm, 5 µm diameter, pore size 100 Å; Phenomenex Scandinavia, Västra Frölunda, Sweden) and a detector (Decade, Kovalent AB, Sweden) operated at 400 mV versus the cell were used. The mobile phase was delivered at 0.3 ml/min and consisted of 58 mM citric acid, 135 mM NaOH, 0.107 mM Na2–EDTA and 20% methanol. The second system consisted of a pump (UltiMate 3000 Pump; Thermo Scientific, Darmstadt, Germany), a reversed phase column (2.0 x 50 mm, 3 µm diameter; pore size 100 Å; Phenomenex Scandinavia, Västra Frölunda, Sweden) and a detector (Dionex, Västra Frölunda, Sweden) operated at 220 mV versus the cell. The mobile phase was delivered at 0.3 ml/min and consists of f 150 mM NaH2PO4, 4.76 mM citric acid, 3 mM sodium dodecyl sulphate, 50 µM EDTA, as well as 10% MeOH and 15% acetonitrile.

Verification of probe placement

The mice were decapitated and the brains were mounted on a vibroslice device (752 M Vibroslice; Campden Instruments Ltd., Loughborough, UK). The brains were cut in 50 µm sections, and the location was determined (Paxinos *et al*, 1998) by observation using light microscopy.

Conditioned place preference (CPP)

For all CPP experiments a 2-chambered conditioned place preference apparatus (45 lux) with distinct visual and tactile cues was used. The experiment consisted of the pre-conditioning phase (day 1), conditioning phase (day 2-5) and post-conditioning phase (day 6).

The first CPP test was conducted to evaluate the effect of sCT on the rewarding properties of alcohol. The mice at the pre-conditioning phase were placed in the apparatus with free access to both compartments for 20 minutes, in order to determine the initial place of preference. Conditioning (20 minutes per session) was done using a biased procedure in which alcohol (1.75 g/kg, IP) was paired with the least preferred compartment and vehicle (saline solution, IP) with the preferred compartment. In these experiments, sCT (5 µg/kg, IP) or vehicle (saline solution, IP) was administered 30 minutes prior to alcohol or vehicle (saline solution, IP) administration on each of the four conditioning days, creating treatment groups of vehicle-alcohol and sCT-alcohol. The injections were altered between morning and afternoon in a balanced design. At post-conditioning, the mice were untreated and were placed in the middle of the

chamber, with free access to both compartments for 20 minutes. In a control experiment for sCT, separate mice were subjected to the exact same procedure, but received vehicle (saline solution, IP) injections instead of alcohol in combination with sCT (5 µg/kg, IP) or vehicle (saline solution, IP) throughout the conditioning (non-alcohol conditioned control group), creating the following treatment groups: vehicle-vehicle and sCT-vehicle).

The second experiment was conducted in a separate mice group and was designed to investigate the effect of sCT on memory consolidation of alcohol reward. In this experiment, mice were injected with vehicle (saline solution, IP) at the pre-conditioning phase and were placed in the chamber with free access to both compartments during 20 minutes in order to determine the initial place of preference. Conditioning (20 minutes per session) was done using a biased procedure in which alcohol (1.75 g/kg, IP) was paired with the least preferred compartment and vehicle (saline solution, IP) with the preferred compartment. All mice received one alcohol and one vehicle injection every day and the injections were altered between morning and afternoon in a balanced design. At post-conditioning, mice were injected with sCT (5 µg/kg, IP) or with an equal volume of vehicle solution (saline solution, IP) and 30 minutes later placed on the midline between the two compartments with free access to both compartments for 20 minutes (creating the following treatment groups; alcohol-vehicle and alcohol-sCT). In a second control experiment for sCT, separate mice were subjected to the exact same procedure, but received vehicle (saline) injections instead of alcohol throughout the conditioning (non-alcohol conditioned control group) and sCT (5 µg/kg, IP) or with an

equal volume of vehicle solution (saline solution, IP) at post-conditioning, creating the following treatment groups: vehicle-vehicle and vehicle-sCT.

In all experiments the injections were altered between morning and afternoon in a balanced design and with equal number of animals between groups. CPP was calculated as the difference in % of total time spent in the drug-paired (i.e. less preferred) compartment during the post-conditioning and the pre-conditioning sessions.

REFERENCES

Bassareo V, Di Chiara G (1999). Differential responsiveness of dopamine transmission to food-stimuli in nucleus accumbens shell/core compartments. *Neuroscience* **89**(3): 637-641.

Braegger FE, Asarian L, Dahl K, Lutz TA, Boyle CN (2014). The role of the area postrema in the anorectic effects of amylin and salmon calcitonin: behavioral and neuronal phenotyping. *The European journal of neuroscience* **40**(7): 3055-3066.

Cadoni C, Solinas M, Di Chiara G (2000). Psychostimulant sensitization: differential changes in accumbal shell and core dopamine. *European journal of pharmacology* **388**(1): 69-76.

Clarke RB, Adermark L, Chau P, Soderpalm B, Ericson M (2014). Increase in nucleus accumbens dopamine levels following local ethanol administration is not mediated by acetaldehyde. *Alcohol Alcohol* **49**(5): 498-504.

Egecioglu E, Steensland P, Fredriksson I, Feltmann K, Engel JA, Jerlhag E (2013). The glucagon-like peptide 1 analogue Exendin-4 attenuates alcohol mediated behaviors in rodents. *Psychoneuroendocrinology* **38**(8): 1259-1270.

Jerlhag E, Grotli M, Luthman K, Svensson L, Engel JA (2006). Role of the subunit composition of central nicotinic acetylcholine receptors for the stimulatory and dopamine-enhancing effects of ethanol. *Alcohol Alcohol* **41**(5): 486-493.

Lutz TA, Tschudy S, Rushing PA, Scharrer E (2000). Amylin receptors mediate the anorectic action of salmon calcitonin (sCT). *Peptides* **21**(2): 233-238.

Mietlicki-Baase EG, Rupprecht LE, Olivos DR, Zimmer DJ, Alter MD, Pierce RC*, et al* (2013). Amylin Receptor Signaling in the Ventral Tegmental Area is Physiologically Relevant for the Control of Food Intake. *Neuropsychopharmacology* **38**(9): 1685-1697.

Paxinos G, Watson C (1998). *The brain stereotaxic coordinates* Academic Press: New York.

Pecile A, Guidobono F, Netti C, Sibilia V, Biella G, Braga PC (1987). Calcitonin gene-related peptide: antinociceptive activity in rats, comparison with calcitonin. *Regulatory Peptides* **18**(3–4): 189-199.
